# Physical performance analysis: A new approach to assessing free-living physical activity in musculoskeletal pain and mobility-limited populations

**DOI:** 10.1371/journal.pone.0172804

**Published:** 2017-02-24

**Authors:** Matthew Smuck, Christy Tomkins-Lane, Ma Agnes Ith, Renata Jarosz, Ming-Chih Jeffrey Kao

**Affiliations:** 1 PM&R Section, Department of Orthopaedic Surgery, Stanford University, Redwood City, California, United States of America; 2 Wearable Health Lab, Department of Orthopaedic Surgery, Stanford University, Redwood City, California, United States of America; 3 Department of Physical Education & Recreation, Mount Royal University, Calgary, Canada; 4 Department of Anesthesia and Pain Management, Stanford University, Redwood City, California, United States of America; Vanderbilt University, UNITED STATES

## Abstract

**Background:**

Accurate measurement of physical performance in individuals with musculoskeletal pain is essential. Accelerometry is a powerful tool for this purpose, yet the current methods designed to evaluate energy expenditure are not optimized for this population. The goal of this study is to empirically derive a method of accelerometry analysis specifically for musculoskeletal pain populations.

**Methods:**

We extracted data from 6,796 participants in the 2003–4 National Health and Nutrition Examination Survey (NHANES) including: 7-day accelerometry, health and pain questionnaires, and anthropomorphics. Custom macros were used for data processing, complex survey regression analyses, model selection, and statistical adjustment. After controlling for a multitude of variables that influence physical activity, we investigated whether distinct accelerometry profiles accompany pain in different locations of the body; and we identified the intensity intervals that best characterized these profiles.

**Results:**

Unique accelerometry profiles were observed for pain in different body regions, logically clustering together based on proximity. Based on this, the following novel intervals (counts/minute) were identified and defined: Performance Sedentary (PSE) = 1–100, Performance Light 1 (PL1) = 101–350, Performance Light 2 (PL2) = 351–800, Performance Light 3 (PL3) = 801–2500, and Performance Moderate/Vigorous (PMV) = 2501–30000. The refinement of accelerometry signals into these new intervals, including 3 distinct ranges that fit inside the established light activity range, best captures alterations in real-life physical performance as a result of regional pain.

**Discussion and conclusions:**

These new accelerometry intervals provide a model for objective measurement of real-life physical performance in people with pain and musculoskeletal disorders, with many potential uses. They may be used to better evaluate the relationship between pain and daily physical function, monitor musculoskeletal disease progression, gauge disease severity, inform exercise prescription, and quantify the functional impact of treatments. Based on these findings, we recommend that future studies of pain and musculoskeletal disorders analyze accelerometry output based on these new “physical performance” intervals.

## Introduction

Physical inactivity is both a cause and consequence of many musculoskeletal disorders. Accordingly, clinical studies are expected to assess physical function, and report functional outcomes. When considering function, it is important to recognize the two distinct categories, defined by the International Classification of Functioning (ICF): capacity and performance.[1] Capacity represents the capability of a person to complete a given task in a controlled environment (e.g. a timed stair climb or walking test), while performance represents what a person does in his or her current environment. Many measures exist to capture capacity in research and in the clinical setting, while the ability to measure performance remains limited in both settings. Continuous activity monitoring seems a logical means of assessing performance by measuring free-living physical activity; yet, to date only a handful of musculoskeletal studies have employed activity monitors with surprisingly limited results.

Early studies applied pedometers to examine the impact of knee and hip osteoarthritis, low back pain and lumbar spinal stenosis on performance, demonstrating significant differences between disease subjects and controls in daily step counts.[2,3] More sophisticated accelerometers were used in similar populations, producing few additional insights by finding either small or no differences between disease subjects and controls in average daily activity counts and time above validated activity intensity thresholds.[4,5,6] The failure to gain additional insight from accelerometry tempered enthusiasm for activity monitoring research in these populations. Nonetheless, these lackluster results are counterintuitive; after all, limitations in physical performance are a hallmark feature of painful musculoskeletal disorders.[7,8–9] Accordingly, research in the spine field has yielded more promising results using accelerometry,[10,11,12] and there is new optimism that accelerometry can transform clinical research in this field.[13,14]

One recent study in patients with spinal stenosis revealed the usefulness of a single disease specific accelerometry measure.[7] Additionally, recent research using accelerometers has demonstrated empirically, for the first time, that people with painful mobility limitations are extremely sedentary with almost no activity above the established moderate intensity activity threshold,[2,15] while another study showed the importance of small improvements in activity to reduce the risk of obesity-related low back pain.[10] Such findings compelled us to undertake this investigation to optimize the methods of accelerometry analysis in musculoskeletal and pain research, both to improve outcomes assessment and clinical care in these populations.

We suspect that current methods of accelerometry analysis have contributed to the limited findings in studies involving pain and musculoskeletal disorders. The current state of the art in physical activity assessment using accelerometry is driven by cardiovascular and fitness research, as these were the targets of early research using accelerometers. Thus, established methods stratify physical activity as it relates to energy expenditure [in the form of metabolic equivalents and oxygen consumption (VO_2_)] into the following serial activity intensity categories: sedentary, light, moderate, and vigorous.[16] Using this stratification of activity, 90% or more of the average person’s daily non-sedentary activity falls into the light activity range.[4,17] Due to the greater impact of moderate and vigorous activity on cardiovascular disease and fitness, the established signal analysis emphasizes these higher intensity ranges and minimizes activity recorded in the light intensity range. While these methods of signal analysis are appropriate for fitness research, they are not designed or validated to measure the impact of mobility-limiting disorders on a person’s daily physical performance. It is now clear that established methods focusing on moderate and vigorous activity can miss potentially important perturbations in the light activity range,[18] where clinical experience and recent research demonstrates greater impact in musculoskeletal pain populations.[2,5–6,10–11,15,19–22] Thus, it is likely that important relationships between pain and physical activity have been overlooked using the existing methods of activity stratification.

We hypothesize that pain in different regions of the body is associated with distinct impact on free-living physical activity (performance) as measured by an accelerometer. We also hypothesize that by examining these pain-related differences in physical performance, we will reveal methods to optimize investigations of physical performance in painful musculoskeletal disorders. To this end, the goal of this study is to investigate the impact of regional body pain on free-living physical activity in the U.S. population. Specifically, we investigate the 2003–4 NHANES dataset, selected because it includes both 7-day free-living accelerometry and a comprehensive health and pain questionnaire, to determine whether people with reported pain in different regions of the body display divergent accelerometry signals; and if so, to define the intensity intervals that best characterize them.

## Materials and methods

### Software

Functions and programs were written in Python 2.7 (Python Software Foundation, Beaverton, OR) for data processing and pre-computation. Procedures and macros were written in SAS 9.2 (SAS Institute, Cary, NC) for data processing, complex survey regression analyses, model selection, and statistical adjustment. R 2.11 (The R Project for Statistical Computing, Vienna, Austria) was used for clustering and visualization.

### Data description

The National Health and Nutrition Examination Survey (NHANES) is a continuous study conducted by the National Center for Health Statistics, designed to assess the health of children and adults in the U.S. Leveraging the U.S. Census data, NHANES provides survey samples that are representative of the U.S. non-institutionalized population (e.g., excluding the military, imprisoned, or hospitalized population) using a multi-stage, weighted, complex survey design. The NHANES survey is composed of an interview and a physical examination section, performed in mobile examination centers. The present analysis used data from NHANES 2003–2004, selected since it includes a comprehensive bodily pain questionnaire in addition to physical activity monitoring using accelerometers (ActiGraph AM-7164; ActiGraph, Pensacola, FL, USA). A total 6,796 subjects from NHANES (2003–2004) provided sufficient data for inclusion in this analysis based on a validated wear-time analysis [23]. All data were obtained from the National Center for Health Statistics website (http://www.cdc.gov/nchs/).

### Accelerometry data

All subjects were instructed to wear an ActiGraph model 7164 accelerometer (ActiGraph, LLC; Ft. Walton Beach, FL) over the right hip for 7 consecutive days after the examination. Wear-time estimation based on the National Cancer Institute (NCI) protocol is used to calculate the proportion of wear-time the subject spends in that interval, averaged across days with sufficient wear times. Only days with 10 or more hours of valid wear-time, inferred using the NCI algorithm, are retained.[23]

### Analysis

#### Overview

To observe the influence of regional body pain on the accelerometry signals, we simplify the signal intensity scale into defined intervals, then adjust for all demographic, social, and medical variables found to impact these signals in our previous work.[18] From this adjusted data, we uncover the accelerometry profiles that relate to pain in different body regions. Finally, normalization and hierarchical clustering are used to expose the accelerometry intervals that are in tune with alterations in physical performance due to regional body pain. Accordingly, we call this new approach *physical performance* (as opposed to “physical activity”) analysis. Henceforth, the statistical and analytical methods used to achieve this are described in greater detail.

#### Per-subject Motion Intensity Profile (MIP)–Un-adjusted

The accelerometers used in this study measure per-minute motion intensity as an integer taking possible values between 0 and 32,767, inclusively. As described in our previous work on this dataset,[18] this is simplified into the following inclusive intensity intervals: [0], [1,10], [11,20], [21,30], [31,40], [41,50], [51,60], [61,70], [71,80], [81,90], [91,100], [101,110], [111,120], [121,130], [131,140], [141–150], [151–160], [161–170], [171–180], [181–190], [191–200], [201–250], [251–300], [301–350], [351–400], [401–450], [451–500], [501–600], [601–700], [701–800], [801–900], [901–1000], [1001,1500], [1501–2000], [2001–2500], [2501–3000], [3001–3500], [3501–4000], [4001–4500], [4501–5000], [5001–6000], [6001–7000], [7001–8000], [8001–9000], [9001–10000], [10001,15000], [15001–20000], [20001,25000], [25001,30000], [30001,32767]. For each subject, for each interval, the number of minutes recorded across the 7-day period is calculated and converted to a daily average, then log-transformed. For each subject this creates a motion intensity profile (MIP). We label this the “un-adjusted MIP” as we later adjust each subject’s MIP for multiple variables.

#### Self-reported pain and co-occurrence of pain types

Self-reported pain was determined during the NHANES examination by affirmative response(s) to questions regarding experience with pain during the previous 3 months and during the previous year. This included inquiries about pain in the head, face, neck, upper back, lower back, lower back with radiation, chest, abdomen, shoulder, arm, hand, leg, and foot. Table 1 displays the details of these questions and how they were coded into 15 distinct pain types for the purposes of this study. The prevalence of the 15 pain types, ***p***_***j***_, as well as the all-pairwise co-occurrence prevalences, ***p***_***j*,*k***_, were calculated using SAS. The metric for over-representation of co-occurrence between pain type *j* and *k* is defined as: [pj,kpj⋅pk]-1.

**Table 1:** Coding of the 15 different pain types.

The following questions are about pain you may have experienced in the past 3 months. Please refer to pain that lasted a whole day or more.		
**Question**	**Response**	**Item Code**
During the past 3 months, did you have neck pain?	Yes	**NECK3**
During the past 3 months, did you have low back pain?	Yes	**LBP3**
Did this pain spread down either leg to areas below the knees?	Yes	**LBP3R**
During the past 3 months, did you have severe headaches or migraines?	Yes	**HAM3**
Have you had a problem with pain that lasted more 1 month, more than 3 months, or more than 1 year? Subjects who answer affirmative proceed to the following.		
Regarding your pain problem, which regions are affected?		**Item Code**
Head affected		**CPHEAD**
Face/dental affected		
Right shoulder girdle affected		**CPSHDR**
Left shoulder girdle affected		
Right upper arm affected		**CPARM**
Left upper arm affected		
Right mid-arm affected		
Left mid-arm affected		
Right lower arm affected		
Left lower arm affected		
Right upper back affected		**CPUBACK**
Left upper back affected		
Right lower back affected		**CPLBACK**
Left lower back affected		
Right buttock affected		
Left buttock affected		
Spine affected		
Right upper leg affected		**CPLEG**
Left upper leg affected		
Right mid-leg affected		
Left mid-leg affected		
Right lower leg affected		
Left lower leg affected		
Neck affected		**CPNECK**
Sternum affected		**CPCHEST**
Right chest affected		
Left chest affected		
Abdomen affected		**CPABD**
Right hand affected		**CPHAND**
Left hand affected		
Right foot affected		**CPFOOT**

Coding of the 15 different self-reported pain types. The abbreviations used for each of the 15 different self-reported pain types (right hand column) are shown next to their representative question(s) during the NHANES examination.

#### Creation of the pain adjusted MIPs

Using a regression model, each of the per-subject MIPs is then adjusted for the demographic, anthropomorphic, social, and medical variables found to impact these signals in our previous work [18]. Since this accelerometry data is provided in a minute-by-minute measurement of counts, given intervals of sufficiently wide range, for each interval the number of minutes behaves as a continuous variable. We thus adopt the weight survey multiple linear regression model, for every interval *[a*,*b]* and for every subject *i*:

$$C_{i}^{\left[a,b\right]}=A_{i}^{\left[a,b\right]}+\sum_{d}\theta_{d}^{\left[a,b\right]}\cdot d_{i}+\sum_{t}\theta_{t}^{\left[a,b\right]}\cdot t_{i}^{\left[a,b\right]}+\sum_{m}\theta_{m}^{\left[a,b\right]}\cdot m_{i}^{\left[a,b\right]}+\varepsilon_{i}^{\left[a,b\right]}$$

where *d*’s, *t*’s, and *m*’s represent demographic, anthropometric, and medical variables, respectively; *ε* represents independent and identically distributed error.

Given the estimated parameters, then, the true signal vector is estimated for every interval *[a*,*b]* and for every subject *i*:

$${\hat{A}}_{i}^{\left[a,b\right]}=C_{i}^{\left[a,b\right]}-\sum_{d}{\hat{\theta }}_{d}^{\left[a,b\right]}\cdot d_{i}+\sum_{t}{\hat{\theta }}_{t}^{\left[a,b\right]}\cdot t_{i}^{\left[a,b\right]}+\sum_{m}{\hat{\theta }}_{m}^{\left[a,b\right]}\cdot m_{i}^{\left[a,b\right]}$$

The collection of estimated true signals, ${\hat{A}}_{i}={\left\{{\hat{A}}_{i}^{\left[a,b\right]}\right\}}_{\left[a,b\right]}$, then, constitutes the adjusted MIP for subject *i*.

After the above procedure is performed for all subjects *i* and all intervals *[a*,*b]*, we calculate the average adjusted MIP (pain adjusted MIP) for each regional body pain *P*:

$${\hat{A}}^{p}=\frac{1}{\sum_{i}[P_{i}]}\left[\begin{array}{c} \vdots \\ \sum_{i}\left[P_{i}\right]{\hat{A}}_{i}^{\left[a,b\right]}\\ \vdots \end{array}\right]$$

where *P*_*i*_ is an indicator variable representing if subject *i* has the particular regional body pain. This procedure is repeated for each regional body pain.

#### Normalization and clustering

Two-way hierarchical clustering was performed across body regions *P* and across intensity intervals *[a*,*b]*. The log-transformed pain adjusted MIP is normalized within each intensity interval and across each regional body pain. Similarity between vectors was measured using the Euclidean distance metric. Hierarchical clustering was performed using the average-linkage method.

## Results

### Pain types co-occur as a function of proximity

We first study the relationships between the different pain types based on self-reported symptoms alone. Since each subject could report multiple locations of pain, we studied the co-occurrence of the different pains types. Using cluster analysis, pain types that tend to occur in the same subjects will be clustered closer to each other.

Using overrepresentation of co-occurrence as a distance metric, hierarchical clustering of the 15 pain types was performed. The dendrogram is provided in the supporting information (S1 Fig). Closely related clusters include similarly defined regional pain, such as low back pain with radiation (LBPR3) during the last 3 months with chronic pain in low back (CPLBACK) during the past year, as well as spatially similar quadrants of the body, such as chronic pain in the neck (CPNECK) with chronic pain in the upper back (CPUBACK), and low back pain during the last 3 months (LBP3) with chronic pain in the leg (CPLEG) and chronic pain in the foot (CPFOOT).

We note, however, that while interesting, this analysis alone provides little additional information besides offering a logical observation of the co-occurrence of regional pain types. This pattern of co-occurrence may reflect local pain generators, biomechanical linkage, or even poor subject report due to perceived ambiguity of the definition of different body regions.

### Un-adjusted MIPs do not capture pain type signals

Next, we make use of the accelerometry readings in an attempt to reveal similarities between the 15 pain types, and perform hierarchical clustering on the per-subject MIPs. Discarding the extreme ranges, the intervals appear to cluster into 3 wide contiguous ranges ([1,350], [351,3000], and [3001,25000]) with less discrimination and poor correspondence to the widely-accepted VO_2_ derived intensity intervals ([0,100] sedentary, [101,1952] light, [1953,5724] moderate, [5725,32767] vigorous).[16]

### Clustering of axial versus appendageal pain with the pain adjusted MIPs

Discretized multivariate adjustment model (DMAR), described previously,[18] is applied to adjust for the population effects of demographic, anthropomorphic, social, and medical variables. These adjusted MIPs are then used for further analyses.

**Fig 1:**
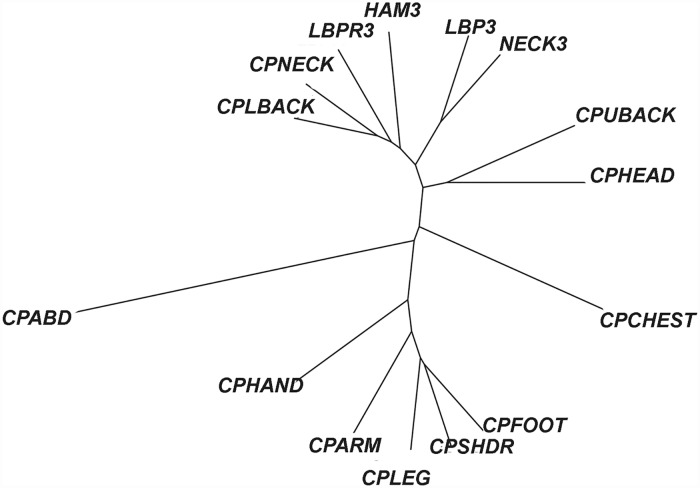
Clustering of pain types using adjusted MIPs. In a dendrogram, objects similar to each other are arranged close to each other. Their relative distance is represented by the height of the lowest branch that joins, directly or indirectly, to the corresponding leaves of the tree. After adjusting the MIPs (“adjusted MIPs”) for population effects of several demographic, anthropomorphic, social, and medical variables, this dendogram shows hierarchical clustering of the 15 different pain types. Interestingly, across body regions, the dendrogram reveals close clustering of axial pain in contrast to appendageal pain. Abdominal pain and chest pain are also distinguished from the axial pain and appendageal pain clusters, and from each other. The definitions of the 15 different pain type abbreviations are provided in Table 1.

Hierarchical clustering of the 15 different pain types, using the adjusted MIPs, is displayed in a dendrogram in Fig 1. Interestingly, across body regions, there is close clustering of axial pain in contrast to appendageal pain, and vice versa. While abdominal pain and chest pain are distinguished from both the axial pain and appendageal pain clusters, and from each other.

### Discovery of novel intervals

**Fig 2:**
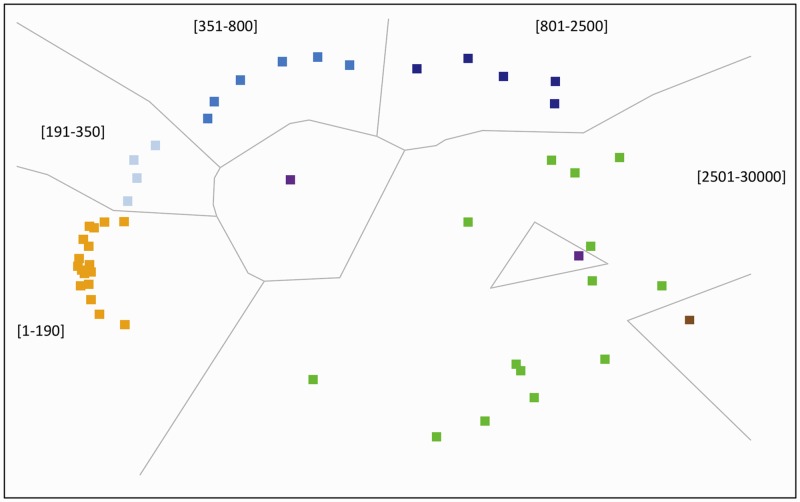
Multi-dimensional scaling of the pain adjusted MIPs. The figure shows the coherent tight clustering of counts per-minute intervals within certain ranges. There is clear segregation of the accelerometry signal into logically coherent and numerically contiguous intervals: yellow (1–190), light blue (191–350), medium blue (351–800), dark blue (801–2500), and green (2501–30000). Extremes values in the low range (brown, 0) and high range (purple, >30000) are not considered.

**Fig 3:**
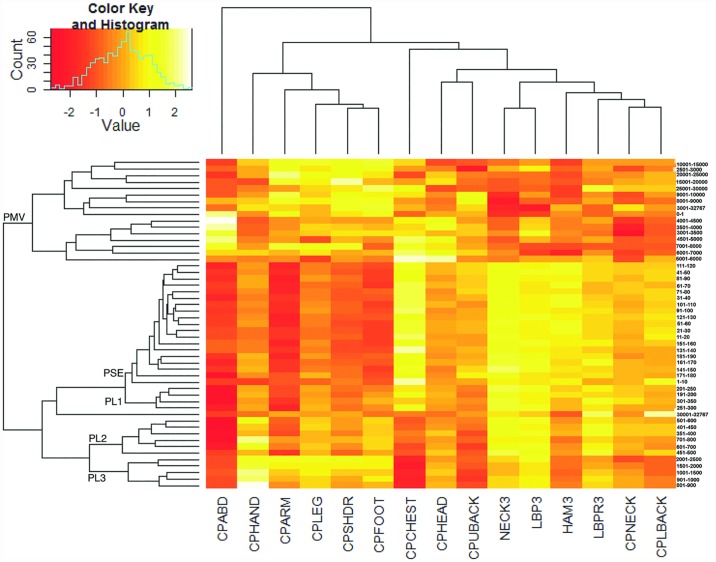
Two-way hierarchical clustering reveals new intervals. The logically coherent and numerically contiguous intervals described in Fig 2 are adopted here in a two-way hierarchical clustering structured to define intensity intervals that are tuned to the presence of regional pain. The figure demonstrates the relationship between the clustering of axial pain and appendageal pain (bottom row) and the segregation of the accelerometry intervals (right hand column). The ordering of the columns and rows is not selected based on activity intensity, rather it is selected to optimize relationships and segregations between the many cells as visualized by the resulting heatmap. This reveals 5 distinct clusters of continuous activity intensity (excluding the extreme values of 0 and >30000) that define our new intervals (left hand column): Performance Sedentary (PSE) = 1–100, Performance Light 1 (PL1) = 101–350, Performance Light 2 (PL2) = 351–800, Performance Light 3 (PL3) = 801–2500, and Performance Moderate and Vigorous (PMV) = 2501–30000.

Across activity intensity intervals, there is coherent tight clustering of counts per-minute intervals within certain ranges, which are adopted next to define intensity intervals that are tuned to the presence of regional pain. More extreme intensities in the low end (zero) and high end (>30,000) are not considered. We discover that using this method, certain ranges of the accelerometry signal tend to cluster together in logically coherent and numerically contiguous patches (Fig 2). A similar pattern is discovered in two-way hierarchical clustering (Fig 3).

Based on this clustering of contiguous intervals, we construct novel thresholds for analysis of accelerometry in pain and musculoskeletal research. Thus, the following intervals are defined: Performance Sedentary (PSE) = 1–100, Performance Light 1 (PL1) = 101–350, Performance Light 2 (PL2) = 351–800, Performance Light 3 (PL3) = 801–2500, and Performance Moderate and Vigorous (PMV) = 2501–30000. By construction, these intervals are optimized to distinguishing alterations in physical performance as a function of regional body pain. Accordingly, we recommend calling them the “*physical performance intervals*.”

Compared to the intervals derived from VO_2_ measures, and reflected in the names we selected, we find that the PSE interval grossly corresponds to the “sedentary” range; PL1, PL2, and PL3 correspond to the “light” range; and PMV corresponds to the “moderate” and “vigorous” ranges. These results align with our intuitive expectation, that for the purpose of musculoskeletal pain the light activity range is most informative. In fact, these results show there is a gradient of “light” activities that can be usefully refined into 3 subcategories for musculoskeletal and pain research. As shown here, these subcategories are able to better capture alterations in real-life physical performance as a result of regional pain.

## Discussion

### Background and rationale for this study

Free-living physical activity (performance) is a marker of overall health and has well-defined relationships to many diseases. People with musculoskeletal disorders report performance limitations due to pain, and clinicians consider alterations in free-living activity a key marker of the underlying disease. For this reason, functional outcomes are generally recognized as the principal clinical variable to study in musculoskeletal disorders. As detailed in the introduction, there are two components of function: capacity and performance. Accurately measuring performance in patients with painful musculoskeletal disorders is likely of greater value than measuring capacity, given that patients typically complain of limitations in performance, not capacity. However, accuracy in measuring performance in this population remains limited by reliance on self-reported measures.

**Fig 4:**
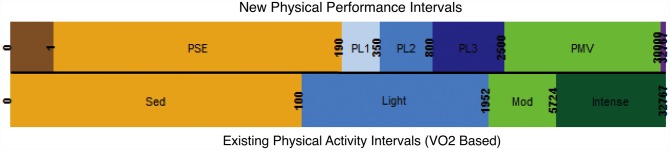
Comparison of new and established intervals. A side-by-side comparison of the established VO_2_ -derived intervals that measure physical activity (bottom row) alongside the new pain-derived intervals that measure physical performance (top row) as uncovered in Fig 3.

Accelerometers are able to objectively record real-life physical activity, so it is surprising that their use in musculoskeletal research remains undefined. Despite the importance of physical function and pain in musculoskeletal disease research, few studies have examined the relationship between these constructs. Using accelerometry, we were able to investigate this relationship and empirically-derive new intervals (“physical performance intervals”) optimized for the analysis of accelerometry data from people with musculoskeletal pain. Interestingly, portions of these new intervals fell close in line with the existing VO_2_ based measures (Fig 4). More interesting, these new intervals provide greater discrimination in the light activity range, corresponding with the types of activities that are commonly problematic for patients with painful musculoskeletal disorders.

Prior to this investigation we hypothesized that the existing accelerometry thresholds were not optimized for insight into the altered real-life physical performance of people with musculoskeletal pain. This hypothesis stemmed from three insights we gained from our prior work. Specifically, our earlier large-scale analysis of physical activity in the US population revealed that many strong effects of physical activity can be seen at light intensities, but these effects are masked within the intervals stemming from the traditional VO_2_ cut-points.[18] Second, our investigation into the role of physical activity in the relationship between back pain and obesity found that the mid-range of physical activity has a robust influence, not higher intensity activities.[10] This suggests that mechanisms other than fitness and energy expenditure underlie physical activity’s influence on the connection between back pain and obesity. Third, a study of patients with spinal stenosis revealed the usefulness of a single accelerometry measure customized to this population.[7] This study supported the hypothesis that disease-specific metrics may be needed when analyzing accelerometry data. Together, these discoveries signify a different approach is necessary to characterize the impact of musculoskeletal pain on accelerometry measurements, which this study provides.

A key finding of this study is the importance of light-range activity in musculoskeletal pain since the results indicate that pain influences the accelerometer signal here more than in the sedentary, moderate or vigorous ranges. Recent work by others has also highlighted the need for an improved focus on light intensity activity in pain and mobility-limited populations. For example, research in osteoarthritis has demonstrated that both sedentary behavior and time spent in light physical activity are related to disability, risk for disability, and physical capacity, independent of time spent in moderate to vigorous physical activity.[5,8–9,19,24–26] This is important because these findings highlight a potential new focus on sedentary behavior and light intensity activity (in contrast to the current focus on moderate and vigorous physical activity). A focus on light activity is also supported by research on the relationship between physical activity and health. In a recent review, Powell et al. examined a dose-response curve for volume of activity and all-cause mortality.[27] Although this curve used mortality, and not a pain specific outcome, some interesting concepts are derived from examining this curve. First, there was no lower threshold for benefits, as reductions in risk begin with any activity above sedentary, supporting the notion that some activity is always better than none.[28] This is encouraging for pain and mobility-limited populations because it demonstrates the potential for health benefits from small but achievable improvements in activity. Second, while adults generally do not achieve the current recommended amount of moderate intensity activity, the greatest benefits for health and function were found for increments in activity within the light range.[27] Thus, people who are unable to meet the current physical activity guidelines can hope to achieve some health benefits through improvements in light activity. This is further supported by a recently published analysis from the English Longitudinal Study of Ageing that showed physical activity of a lower intensity may provide worthwhile health benefits for physically inactive adults.[29] Given that the vast majority of recorded daily physical activity is in this lower end of the activity spectrum, especially for people with mobility limitations, better understanding the light range of activity is essential to understanding patterns of physical performance.[5,20] Our results highlight the importance of light activity by revealing 3 subcategories of light activity that are germane to populations limited by musculoskeletal pain.

### Significance of these findings

The primary innovation of this study is the definition of the novel accelerometry intervals optimized for pain and musculoskeletal research (physical performance intervals). This framework creates new opportunities to investigate the utility of accelerometry in this expansive patient population, and it provides a model for an objective and quantitative functional outcomes instrument that measures real-life physical performance.

To better understand the potential impact of this innovation it is important to review the existing standards for measuring physical function in musculoskeletal research. Currently, objective assessments of function are largely relegated to the research laboratory, usually testing a single dimension of capacity and not physical performance. Thus, for research and for clinical purposes, physical function is almost solely measured using a variety of self-reported outcomes. Unfortunately, these questionnaires contain all of the usual limitations of self-report including: subjectivity, poor discrimination across the disease spectrum, ceiling and floor effects, responder burden, and recall bias.

In addition to the advantages of providing a universal, quantitative and objective measure of real-life physical performance for future investigations, there are a number of potential useful applications for these new physical performance intervals. First, a better understanding of the relationship between pain and physical activity can inform treatment, rehabilitation, and exercise prescription. For example, consider the impact of a more accurate assessment of the types and quantities of real-life physical activity that are beneficial and those that are detrimental in early knee osteoarthritis. Second, by comparing free-living performance to subjective measures of function we may uncover cases where self-reported measures fail to capture the degree of inactivity, and therefore fail to identify true disease risk. Third, observing a patient’s current physical performance as it relates to pain can elucidate practical 'starting points' for precision exercise prescription. For example, if people with a certain musculoskeletal pain (e.g. osteoarthritic knee pain) tend to be most active in the low intensity ranges, then it follows that exercise prescription for moderate intensity exercise may not be practical at the outset. Fourth, accelerometry may also be used to determine the impact of changes in activity within different subgroups of musculoskeletal disorders, and help define the optimal intensity, duration and frequency (dose response) of exercise for people with the disorder.

Finally, it logically follows that accelerometry may be used to monitor disease progression and the effects of treatment. By understanding the relationships between musculoskeletal pain and activity, normalized treatment response curves may be defined in order to track and detect those who are falling behind and need additional care. Passive tracking of a disease-related deterioration over time may contribute to the development of clinical decision-support tools to optimize the timing of musculoskeletal interventions, such as knee arthroplasty. Plus, there is potential for application of these novel intervals in the diagnosis of various musculoskeletal conditions. For instance, emerging patterns in physical performance related to specific pain reporting may contribute to future diagnostic algorithms.

### Study strengths and weaknesses

The greatest strength of this study is that it stems from a large and rigorously acquired population-based dataset. Another strength is our use of custom programs and macros for data processing and a robust statistical analysis. With access to this large database, using our custom programs we were able to control for a multitude of variables that influence physical activity. Thus, we were able to observe the impact of different regional body pains on real-life physical activity. This allowed us to answer our original research questions and achieve our goal. Specifically, we observed physical performance profiles that accompany pain in different body regions, and we uncovered accelerometry intervals that best characterize these profiles. As a result, we were able to empirically derive a novel method of accelerometry analysis for future investigations of physical performance in people with pain and musculoskeletal disorders.

Furthermore, we think the intuitive results provide initial construct validation of our findings. Specifically, the unique pain adjusted MIPs of the different regional pain types clustered together based on proximity. As one would expect, the accelerometry profile of one appendageal pain was more similar to another appendageal pain than it was to pain in the axial region, and vice versa. Additionally, the empirically-derived intervals increase the granularity of physical activity analysis in the light activity range, where clinical experience suggests pain and musculoskeletal disorders have the greatest impact.

Estimates of free-living physical activity can vary between monitors, and different algorithms applied to data from the same monitor can produce different results. Thus, monitor reliability and algorithm validity are important details to consider. This study evaluates data from the ActiGraph monitors used in NHANES, selected in part for their established validity.[23] Since then, ActiGraph monitors have been further validated for their accuracy estimating energy expenditure,[30] and have demonstrated reliability in the assessment of free-living physical activity in adults,[31–34] including those with musculoskeletal pain.[35]

As always, there are weaknesses. Using an existing dataset limited our assessment of regional body pain to the measures provided in the dataset. For instance, we cannot stratify these findings based on the severity of pain, as this was not provided. While we performed a robust analysis of multiple variables, other health-related or demographic variables that were not analyzed or identified in our previous analysis,[18] and therefore not included here, might have an impact on physical activity that may influence our findings. Using an existing dataset also limits our accelerometry data analysis. Specifically, the accelerometry data in this study was available only in 1-minute epochs, so future investigations using these new intervals should evaluate their data at this same sampling rate. Additional studies are required to further validate and define the utility of the novel intervals reported here. Ultimately, these findings support our hypothesis that previous accelerometry studies of patients with musculoskeletal diseases were limited by existing methods of accelerometry analysis. To further test this hypothesis and validate these findings we have initiated prospective clinical studies of patients before and after surgical correction and other interventions for various musculoskeletal disorders.

### Recommendations for future research

We wish to emphasize that these findings are not a criticism of the existing accelerometry thresholds. To the contrary, this study is further evidence that accelerometry analyses should stem from a logical framework. The existing thresholds are validated for their purpose—to understand the impact of fitness on human health. They have proven useful time and again in a multitude of studies involving a wide range of health conditions.[36] It is our hope that this study will similarly serve as the logical framework to guide the use of accelerometry in the fields of pain and musculoskeletal medicine. Based on these findings, we recommend that future studies of pain and musculoskeletal disorders analyze accelerometry output based on the new intervals described in Fig 4. To avoid confusion with the existing thresholds that measure “physical activity”, we recommend using the phrase “physical performance” to describe use of the new intervals reported here.

## Supporting information

S1 Fig Un-adjusted clustering of pain types. In a dendrogram, objects similar to each other are arranged close to each other. Their relative distance is represented by the height of the lowest branch that joins, directly or indirectly, to the corresponding leaves of the tree. Using over-representation of co-occurrence as the distance metric, this dendogram shows that the 15 different types of self-reported pain tend to cluster as a function of proximity. This analysis alone provides little additional information besides offering a logical observation of the co-occurrence of regional pain types. The definitions of the 15 different pain type abbreviations are provided in Table 1.
(TIFF)
