# Supplementary figures and images for: Physical performance analysis: A new approach to assessing free-living physical activity in musculoskeletal pain and mobility-limited populations
Source: PLoS One. 2017 Feb 24;12(2):e0172804. doi: 10.1371/journal.pone.0172804 (PMC5325560; doi:10.1371/journal.pone.0172804)

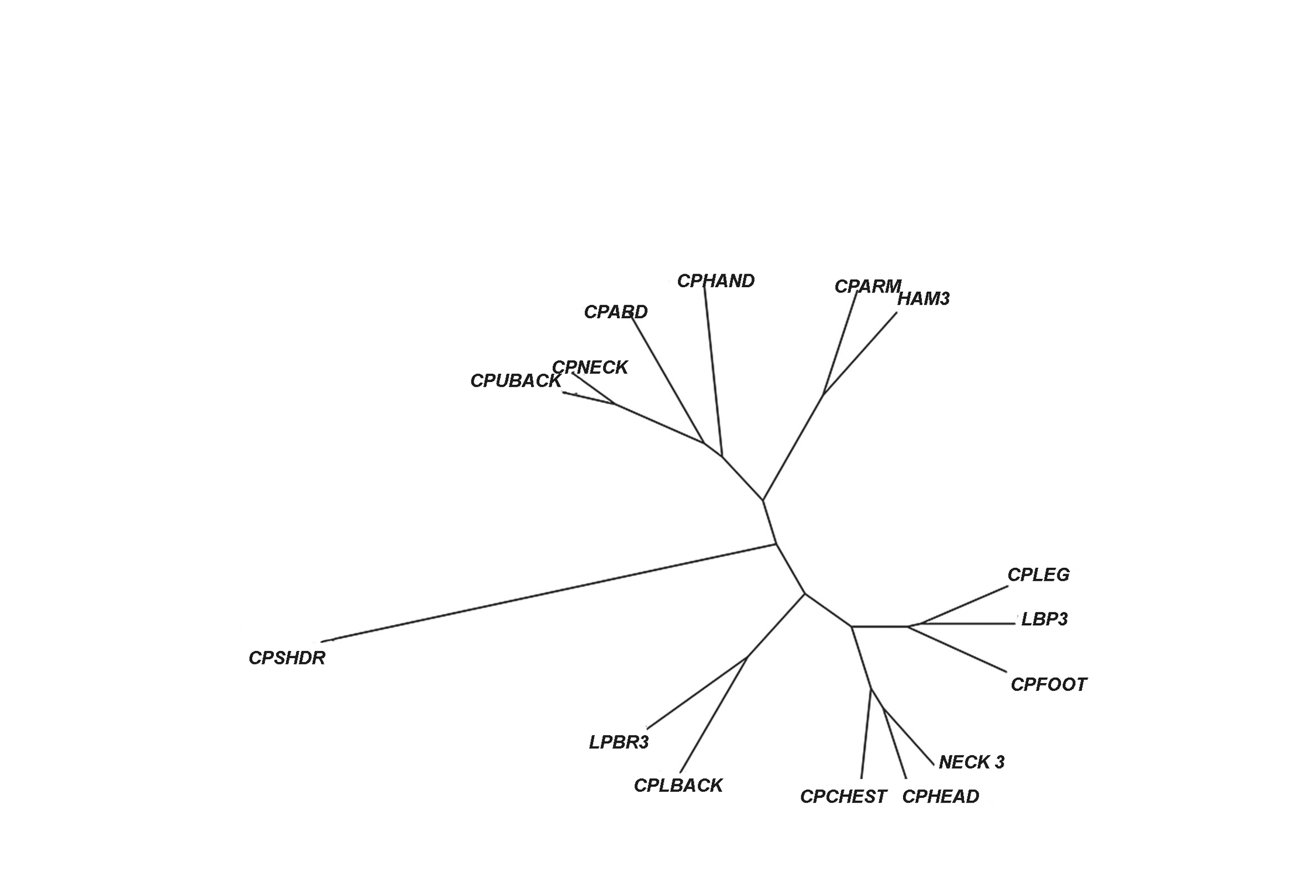

Supplement: S1 Fig — In a dendrogram, objects similar to each other are arranged close to each other. Their relative distance is represented by the height of the lowest branch that joins, directly or indirectly, to the corresponding leaves of the tree. Using over-representation of co-occurrence as the distance metric, this dendogram shows that the 15 different types of self-reported pain tend to cluster as a function of proximity. This analysis alone provides little additional information besides offering a logical observation of the co-occurrence of regional pain types. The definitions of the 15 different pain type abbreviations are provided in Table 1. (TIFF) [file pone.0172804.s001.tiff]
